# Some Syphilinum Cases

**Published:** 1896-03

**Authors:** H. C. Morrow

**Affiliations:** Austin, Texas


### SOME SYPHILINUM CASES.

H. C. Morrow, M. D., Austin, Texas.

### Case I.

Della M. Baby, girl, æt. one year.

Called to see her in September, 1893. Had had summer complaint for several weeks. Symptoms when first seen by myself:

Nausea and vomiting. Peevish, irritable, don't want to be touched or looked at. Stools thin, watery, yellow, very frequent. *Vomits water soon after drinking.* Cutting second tooth. Has only one tooth, and it a stump. Frets as long as a stranger is present. Cries before she passes urine. Vomiting preceded by hacking cough. Becomes pale about the mouth before she vomits. Vomits as if it were thrown out with a gush. Gagging, retching, shows intense nausea and deathly sickness. Vomits milk, curdled. Chewing motion of the mouth when asleep. Very hot fever, day and night, for three weeks. Intense thirst for large quantities of water at frequent intervals. Exceedingly restless, crying and whining; wants to be carried—can only be soothed by carrying up and down the room.

Very much emaciated, old-woman look; skin hangs from limbs in folds. Skin of whole body and face brown, and dirty-looking. Scalp looks as though it had been dried on the skull. Enlarged glands in the neck.

Arsen., Ipec., Phos., Cina, Ant-tart., failed to benefit. Case became desperate. Intensely weak. Has sunk into a stupor. Sleeps with eyes half open. Eyes rolled back in the head. Vomits, vomits, vomits. Stools watery, thin, foul-smelling; very frequent day and night. Rolling head from side to side. Crie cerebrale. Baby unconscious. Automatic jerking of right arm and hand. Sclerotic of both eyes red and congested especially toward inner cauthi. Eyes dim, glazed, corneæ look as though they had been baked. Little lumps of mucus in the eyeballs. Eyeballs dry, no tears. This condition of the eyes is almost always followed by death in a few hours, and such I expected to be termination of this case. Pulse very weak and thready. Anterior fontanelle very much sunken in and scalp over it like leather; no pulsation.

The old ladies predicted death in twelve hours, and I was not prepared to say nay. The baby was an illegitimate child of an adopted daughter, and had been made the legatee of a valuable piece of real estate by its foster grandfather, who had died only a few weeks previously, and whose children naturally were very much incensed on account of this disposition of his property, and who gathered about the dying baby like buzzards keeping vigil over some unfortunate beast in *articulo mortis*. In this dire extremity I turned to Syphilinum, which I administered in the CM potency, Swan, an hour apart, in watery solution. After the very first dose there was a slight improvement. The first dose was given at 2 P. M. By evening there was some cause for encouragement, and by midnight the baby was considerably improved. I then left it, satisfied it would not die during the night, and returned the next morning to find it conscious and so much better that I was able to promise that if it suffered no relapse that it would get well. The jerking of the arm was the first symptom to disappear, as it was the last to come; the rolling of the head went next, and the threatening symptoms gradually subsided, so that in twenty-four hours more there was no longer any doubt.

Its mother, foster grandmother, and a foster aunt were overjoyed at its delivery from imminent death, for they loved the little one dearly, while the remaining relatives looked on me with bitter enmity. The old ladies said it was divine providence, that nothing but the interposition of God could have snatched the baby from the jaws of death.

The medicine was given an hour apart until evening, then two hours until midnight, then three hours until morning, then placebo, and an occasional dose of Syphilinum as I thought it needed. It became, in a few months, a strong, healthy child, and is still living, a monument to the saving virtues of Homœopathy.

---

George H., æt. eighteen. Tall, slender, narrow-chested.

Recently contracted gonorrhœa, for which he took quack nostrums, producing suppression of the discharge. To control resultant fever an allopathic physician was called, to whom the young man denied any venereal infection. Calomel and Quinine were given, which suppressed the fever a few days, when it returned. To me he stated he had chills and fever, for which I prescribed according to the symptoms given; but as I did not get but one-half the story, his fever became continuous, and on examination of his lungs I found a fully-developed pneumonia. To his uncle and then to me he acknowledged having contracted a gonorrhœa. When the discharge ceased the penis back of scrotum became very much swollen and inflamed and sore and tender to touch. The prostate glands became inflamed and very painful. Sensation in rectum of two large, extremely sore lumps, which obstructed the passage, so that it was agonizing to have a stool. Had to sit with legs widely spread apart. Fever constant; increased about 1 P. M., became very high by 4 P. M., and so continued until 4 or 5 A. M. Stupid; incapable of any exertion. Exceedingly nervous and irritable. Sensitive to noise. Does not want to be left alone. Wants light in the room at night; fear in the dark. Afraid of noises which he cannot account for. Fear of mice especially. Cries, and thinks he will not get well. Walking very painful—almost impossible. Has to walk with legs spread wide apart. Pain in rectum, worse from coughing, sneezing, blowing the nose, pressing to stool, motion, standing, walking, and all symptoms worse at night. Very much better by heat and keeping legs spread wide apart. Easiest position lying on back, with legs spread wide apart. Chilliness, even during high fever; worse from draft of air. Dumb chill every other day about 1 P. M. Chilliness up the back; worse when he moves. Hands and feet cold, head hot. Rumbling in abdomen constantly in the P. M. Ears very red; look as if blood would burst out of them. Thinks he is neglected by members of the family, which is not the case. Desire for stool without result. Not satisfied after stool; feels as if more would come. Stools of hard, dry, brown lumps; excruciatingly painful; "would rather die than have a stool," though there is constant desire. Lower lobe of right lung infiltrated and hepatized; dullness on percussion. Hard, racking, tearing cough, which is restrained as much as possible on account of pain in the rectum. Profuse expectoration of bright red blood; a gush of pure blood—in fact, a profuse hemorrhage. The hemorrhages, however, did not ameliorate the violence of the fever. Burning fever; temperature 104° at night; skin burning hot. Has to lie on back on account of pain in the rectum. As inflammation of the lung increased, the pain in the rectum was not so violent, but did not disappear. During increase of heat, languid; hasn't life enough to move; face very red; thirst not marked; great fear that he will not get well. On account of the 4 P. M. agg. and rumbling in the bowels I gave Lycop., then Aconite, and then I learned of the venereal trouble. This determined me to give Syphilinum, which I administered in the CMM potency, Swan. As in the case of the baby, it was given frequently until improvement was well established, and then at less frequent intervals. The effect of the Syphilinum was almost magical. The fever decreased rapidly, the blood disappeared from the expectoration, the hemorrhages ceased, absorption of the exudation took place, and in two days, from having been at the door of death, he was on the highway to recovery. As the lungs became freed from their burden of disease the prostatic and rectal symptoms became more manifest, and these subsiding, were followed by the gonorrhœal discharge being re-established, with entire relief to the deeper-seated internal organs. Nature permitted the blunders of ignorance to be rectified and the young man's life saved.

Hahnemann's teaching, that symptoms disappear in the reverse order of their appearance, was never better illustrated than in this case. It is worthy of remark that this young man at last fell a victim to allopathic dosing, ably assisted by the blunder of an allopathic prescription clerk. Being in a distant town, where there was no homœopathic physician, he was seized with cramping in the bowels, and Strychnia was administered in repeated doses, instead of the less deadly Morphine. The physician, on being called, demonstrated that the fault was not his by giving one of the powders to the family cat, and succeeded in killing it in a few minutes. The whole procedure, blunders and all, was strictly allopathic.

### CASE III.

Francis P. Baby girl, æt. two.

Father had gonorrhœa innumerable times. Baby had frequent attacks of fever, which would continue several days and then pass off. Treatment so far had not been very satisfactory. Fever not very high, but would be followed by very profuse perspiration all over body and limbs. Perspiration smelled musty, cold, clammy. Sometimes the perspiration would be

*only the side that was up*, while the side next the bed would be perfectly dry and hot. Sleepy during the fever, little thirst, waking up at intervals and crying out. Urine very scanty, passed not oftener than twice in twenty-four hours. Red, sandy sediment in urine. Urine almost orange yellow at times.

Syphilinum$^{cm}$, Swan, cured, and the fevers did not return for several months. Recently she had an attack of persistent, continuous fever, and the mother, after trying two homœopaths and one allopath, wrote me about her case. I sent Syphilinum$^{cm}$, Swan, with the same happy result as two years before.

### Case IV.

Stanley G, æt. five.

Tonsils inflamed and swollen and fauces red and inflamed. Deglutition very painful. Yellowish, gray, diphtheritic membrane on both tonsils. Very restless, nervous. Very high fever. Chill every afternoon, about 1 or 2 P. M. Thirst during chill and fever. Face fiery red. Enlarged glands about neck. Sleeps awhile and wakes up crying. Thick, dirty yellow coating on tongue. Profuse salivation. Other remedies did not benefit. Syphilinum$^{cm}$, Swan, gave quick relief and cured.

### Case V.

Mrs. W.

Called in the night to see a large, stout lady, whose husband was a frequent visitor to women of questionable character. The pain came on about 2 P. M.; was located in the abdomen, in the region of the umbilicus. Pain, severe cramping, spasmodic; had to sit up in bed, bent over—apparently a typical Colocynth case. Pains ameliorated by pressure and heat. After waiting two hours for the Colocynth to help, and there being no relief, I began to give Syphilinum. In a short time they commenced to become easier, and in an hour she was asleep. A similar attack a week afterward was also cured by Syphilinum, and that was the last of them. My reason for administering the Syphilinum was that she might have been infected with the gonorrhœal taint from her husband, and the hour at which the attack came on is also very characteristic of this remedy.

